# Optimizing metal/n-AlGaN contact by recessed AlGaN heterostructure with a polarization effect

**DOI:** 10.1039/d2na00813k

**Published:** 2023-03-29

**Authors:** Yuxuan Chen, Ke Jiang, Xiaojuan Sun, Zi-Hui Zhang, Shanli Zhang, Jianwei Ben, Bingxiang Wang, Long Guo, Dabing Li

**Affiliations:** a State Key Laboratory of Luminescence and Applications, Changchun Institute of Optics, Fine Mechanics and Physics, Chinese Academy of Sciences Changchun 130033 China jiangke@ciomp.ac.cn lidb@ciomp.ac.cn; b Center of Materials Science and Optoelectronics Engineering, University of Chinese Academy of Sciences Beijing 100049 China; c Key Laboratory of Electronic Materials and Devices of Tianjin, School of Electronics and Information Engineering, Hebei University of Technology Tianjin 300401 China

## Abstract

With increasing Al mole fraction, n-contact has become an important issue limiting the development of Al-rich AlGaN-based devices. In this work, we have proposed an alternative strategy to optimize the metal/n-AlGaN contact by introducing a heterostructure with a polarization effect and by etching a recess structure through the heterostructure beneath the n-contact metal. Experimentally, we inserted an n-Al_0.6_Ga_0.4_N layer into an Al_0.5_Ga_0.5_N p–n diode on the n-Al_0.5_Ga_0.5_N layer to form a heterostructure, where a high interface electron concentration of 6 × 10^18^ cm^−3^ was achieved with the aid of a polarization effect. As a result, a quasi-vertical Al_0.5_Ga_0.5_N p–n diode with a ∼1 V reduced forward voltage was demonstrated. Numerical calculations verified that the increased electron concentration beneath the n-metal induced by the polarization effect and recess structure was the main reason for the reduced forward voltage. This strategy could simultaneously decrease the Schottky barrier height as well as provide a better carrier transport channel, enhancing both the thermionic emission and tunneling processes. This investigation provides an alternative approach to obtain a good n-contact, especially for Al-rich AlGaN-based devices, such as diodes and LEDs.

## Abstract 2

A convenient and stable strategy is proposed to optimize the contact characteristics of metal/n-AlGaN by introducing a heterostructure with a polarization effect and etching a recess structure through the heterostructure beneath the contact metal.

## Graphical abstract

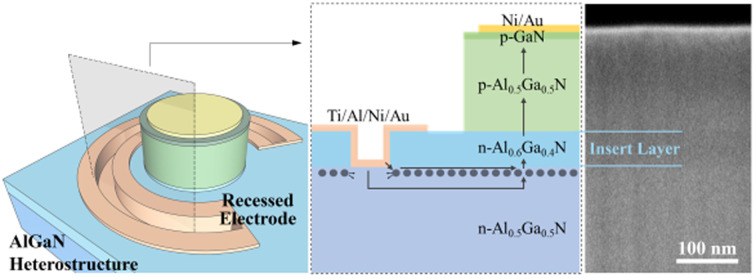

## Introduction

In recent years, great progress has been made in the epi-growth and doping of AlGaN materials, thus boosting the development of AlGaN-based devices.^1–3^ Owing to their superior intrinsic advantages, including a tunable direct wide band-gap from 3.4 to 6.2 eV, high critical electric field, high electron saturation velocity, and outstanding thermal and chemical stability,^4^ AlGaN materials are essential in many photoelectronic and electronic applications, such as ultraviolet lighting,^5^ sterilization,^6^ environmental monitoring,^7^ solar-blind communication,^8^ power switching,^9^ power conversion,^10^ and RF amplifiers.^11^ Although previous reports on AlGaN-based devices have illustrated their irreplaceable characteristics, one among the critical issues preventing further progress is their high-resistance contact. For instance, although the internal quantum efficiency of deep-ultraviolet light-emitting diodes (LEDs) has been propelled up to 80%, the wall-plug efficiency is still lower than 5% because the forward voltage is too high.^12,13^ Another example is AlGaN-based HEMT.^14^ It is also important to reduce the contact resistance for the source and drain regions so that the specific on-resistance can be reduced.^15^ Predictably, the large contact resistance for both optoelectronic and electronic devices also raises the forward voltage and increases the switching losses, especially when the devices are operated at high frequencies. Moreover, the Joule heat will further reduce the device reliability and lifetime.

Therefore, great efforts have been made to reduce the contact resistance and increase the current injection for Al-rich AlGaN-based devices, especially for p-type contacts. For example, a heavily doped p-GaN layer is usually capped as the top p-contact layer in such devices.^16^ However, in such vertical devices, introducing an n-GaN layer as the n-contact layer can be a complex process, thus making a good n-contact on the Al-rich AlGaN layer is very difficult. First, the electron affinity difference between the cathode metal and Al-rich n-AlGaN layer is usually ∼2 eV, which will induce a high Schottky barrier and deplete electrons at the metal/n-AlGaN interface.^17^ When the device is under forward bias, the electron depletion effect at the n-AlGaN surface will become even more significant as the Schottky barrier is reversely biased in this case. Second, the donor activation efficiency in Al-rich AlGaN is very low because of the increased ionization energy.^18^ Lastly, the plasma-caused damages during the fabrication process usually act as deep-level compensation centers in Al-rich AlGaN layers, thus moving the Fermi level away from the conduction band edge and increasing the Schottky barrier height.^19,20^ Therefore, it is necessary to develop a strategy to improve the n-contact characteristics on Al-rich n-AlGaN layers.

For a long time, the conventional approach of using Ti or its alloys has been applied to the n-contact, in which a rapid thermal annealing is generally required to generate the donor-like *V*_N_ on the surface to increase the near surface electron concentration.^21,22^ Recently, several additional efforts have been taken to optimize the contact on the Al-rich n-AlGaN layer, including utilizing alternative metal stacks, optimizing the contact surface, and designing layered structures. For instance, Au-free Ti/Al/Ti/W stacks with low-thermal annealing can reduce the oxidation-induced deep surface states,^23^ while V/Al-based stacks can assist the generation of a heavily n-doped thin layer at the metal–semiconductor (MS) interface,^24^ benefiting the n-contact. Otherwise, a two-step etching process or a NH_3_, SiH_4_, N_2_ mixed-gas pre-treatment of the etched n-AlGaN surface may be used to smooth the n-contact surface or to provide Si and N atoms to occupy the vacancies, respectively.^25,26^ Besides, a thin insulator layer may be grown beneath the metal to form a metal–insulator–semiconductor structure, for sharing the bias, and thus suppressing the surface electron depletion, which can favor the electron intraband tunneling efficiency.^27^ Another way to achieve better metal/n-AlGaN contact is to use a sharply graded n^++^-AlGaN contact layer to obtain a high electron concentration near the interface.^28^ Although those methods optimize the contact a lot, there is still some room for improvement with the increasing Al components.

In this work, we have proposed a more convenient and stable strategy to effectively improve the contact characteristics of the metal/n-AlGaN contact layer by introducing a heterostructure with a certain polarization effect and by etching a recess structure through the heterostructure beneath the n-contact metal. We also experimentally demonstrated the concept in an Al_0.5_Ga_0.5_N-based p–n diode. First, a thin n-Al_0.6_Ga_0.4_N layer was inserted into a p-Al_0.5_Ga_0.5_N/n-Al_0.5_Ga_0.5_N diode on the n-Al_0.5_Ga_0.5_N layer to form an n-Al_0.6_Ga_0.4_N/Al_0.5_Ga_0.5_N heterostructure, whereby an increased electron concentration of 6 × 10^18^ cm^−3^ was achieved by the polarization effect. Then, at the cathode region, the inserted n-Al_0.6_Ga_0.4_N layer was fully etched to form a recess structure. This served as a window for the cathode metal deposited on the n-Al_0.5_Ga_0.5_N layer. As a result, a quasi-vertical Al_0.5_Ga_0.5_N p–n diode with a ∼1 V reduced forward voltage was achieved. Numerical calculations confirmed that the heterostructure polarization effect induced a high concentration of electrons near the interface and this was the main reason for the good results. This strategy could not only provide a good conductive channel to inject electrons, but also could promote the electron concentration near the recess region and lower the Schottky barrier. This investigation provides an alternative approach to obtain a good n-contact, especially for Al-rich AlGaN-based devices, such as diodes and LEDs.

## Experimental section

### Material growth

Here, p–n diode wafers were grown on c-oriented high-temperature-annealed AlN/sapphire substrates by metal–organic chemical vapor deposition (MOCVD). First, a periodic AlN/AlGaN superlattices was grown on the substrate for stress relaxation and dislocation blocking. Trimethyl gallium (TMGa), trimethyl aluminum (TMAl), and ammonia (NH_3_) were used as the Ga, Al, and N precursors, and hydrogen (H_2_) was used as the carrier gas, respectively. The proposed Al content of each AlGaN layer could be obtained by tuning the Al/Ga source ratio. Then, two types of wafers were grown. For the wafers of Devices A and B (Wafer I), an n-Al_0.5_Ga_0.5_N layer of 1 μm, an n-Al_0.6_Ga_0.4_N layer of 60 nm, and a p-Al_0.5_Ga_0.5_N layer of 150 nm were successively grown. For the wafer of Device C (Wafer II), all the parts were the same as for Wafer I except that there was no n-Al_0.6_Ga_0.4_N layer. In particular, SiH_4_ and Cp_2_Mg were used as the Si source and Mg source for the n-type and p-type doping, respectively. Next, a 20 nm heavily doped p-GaN cap layer was deposited on the top of p-AlGaN to form the contact layer. After the epitaxial growth, the wafers were annealed at 900 °C in N_2_ to activate the Mg acceptors.

### Device fabrication

To fabricate the p–n diodes, standard semiconductor device fabrication technology was applied. At first, a SiO_2_ mask of 500 nm was grown by plasma-enhanced chemical vapor deposition (PECVD). Then, photolithography and inductive coupling plasma (ICP, Oxford PlasmaPro 100) etching were employed to pattern and form the mesa structures. In particular, Device A was etched by ∼175 nm to expose the n-Al_0.6_Ga_0.4_N layer; while Devices B and C were etched by ∼230 nm and ∼170 nm, respectively, to expose the n-Al_0.5_Ga_0.5_N layers. Further, as an additional step for Device A, we performed a second pattern transfer and ICP etch to realize a ∼60 nm deep annulus recess around the mesa structure and to expose part of the n-Al_0.5_Ga_0.5_N layer. After removing the residual SiO_2_ mask by buffered oxide etching solution, the devices were immersed in 10% NaOH solution at 90 °C for 10 min to remove the etching defects in the sidewall. Lastly, metal stacks of Ti/Al/Ni/Au (70/130/30/70 nm) were successively deposited as n-electrodes on the n-AlGaN surfaces by photolithography, electron beam evaporation, thermal evaporation, and lift-off processes, and then annealed in a N_2_ atmosphere at 600 °C for 30 s in a rapid thermal annealing system. Similarly, p-electrodes of Ni/Au (30/70 nm) stacks were subsequently deposited on the p-GaN mesas by the same processes and annealed at 550 °C for 5 min.

### Characterizations

Cross-sectional images of the epitaxial wafers and the top-view image of the single microstructure devices were obtained by scanning electron microscopy equipment (SEM, Hitachi S-4800). The surface morphology of the devices and the profile of the recess were measured by atomic force microscopy (AFM, Bruker Multimode 8). The structural parameters and crystalline qualities were estimated through point-focused high resolution X-ray diffraction (HRXRD) measurements performed on a Bruker D8 DISCOVER system equipped with the Cu Kα_1_ radiation line at 0.15406 nm wavelength. To analyze the strain state of the layers and evaluate their in-plane and out-of-plane lattice parameters, reciprocal space mappings (RSMs) for the asymmetrical (−105) diffraction planes were measured. Capacitance–voltage (*C*–*V*) measurements of the n-type Al_0.6_Ga_0.4_N/Al_0.5_Ga_0.5_N heterostructure on the substrate were performed on an SSM495 system at 10 kHz with a small voltage signal of 30 mV. The typical current–voltage (*I*–*V*) curves of each p–n diode were measured using a PDA FS-Pro 380 semiconductor analyzer. All the tests were performed at room temperature (RT) and the experimental data showed good consistency in multiple measurements.

### Simulations

To confirm the physical mechanisms, numerical calculations for the p–n diodes in this work were carried out *via* the commercial software APSYS. In the simulation, the thickness of the n-Al_0.6_Ga_0.4_N layer was set as 50 nm, and the width and depth of the recess were 40 μm and 50 nm, respectively. The distance between the mesa and the recess was 40 μm. The polarization level was set to 40%. The energy band offset for the Al_0.6_Ga_0.4_N/Al_0.5_Ga_0.5_N heterojunction was set to 50/50.^29^ The carrier characteristics and transportation properties in the p–n diodes were simulated by self-consistently solving Schrödinger's equation, Poisson's equation, the current continuity equation, and the drift-diffusion equation with proper boundary conditions using the finite element method.

## Results and discussion

**Fig. 1:**
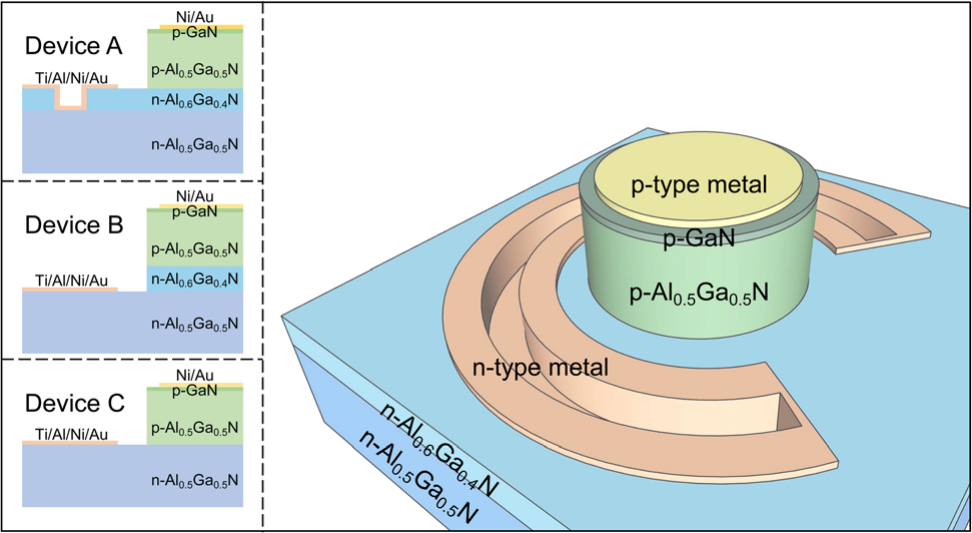
Illustrations of the AlGaN p–n diode. The insets are the cross-sectional structure diagrams of Device A with both heterostructure and recess structure, Device B with just heterostructure, and Device C without heterostructure and recess structure.

Generally, to improve the MS contact characteristics, it is necessary to increase the carrier concentration near the interface to lower the Schottky barrier height and narrow the depletion region. To increase the electron concentration, a c-oriented Al_*x*_Ga_1−*x*_N/Al_*y*_Ga_1−*y*_N (*x* > *y*) heterostructure with polarization-induced local high electron concentration at the interface is proposed. The polarization refers to a specific characteristic of III-nitride materials, consisting of a spontaneous and piezoelectric polarization effect. It comes from the lack of inversion symmetry between the anion and cation positions in the wurtzite unit cell and the lattice mismatch-induced in-plane biaxial stress. Particularly, with increasing the Al content, the concentration will be even more evident, due to the enhanced polarization-induced electron confinement at the heterointerface and the significant screening effect of phonon scattering.^30^ In addition, to keep the high concentration electrons at the interface, as well as to ensure the metal electrode contact with the high concentration electrons at the interface, a recess structure also needs to be fabricated. A quasi-vertical Al_0.5_Ga_0.5_N p–n diode was fabricated to demonstrate our concept. Fig. 1 presents the schematic illustration of the Al_0.5_Ga_0.5_N p–n diode with the recessed Al_0.6_Ga_0.4_N/Al_0.5_Ga_0.5_N heterostructure (Device A). To investigate the influences of the heterostructure and the recess structure on the diode performances, p–n diodes with just the heterostructure (Device B) and without the heterostructure or recess structure (Device C) were also fabricated.

**Fig. 2:**
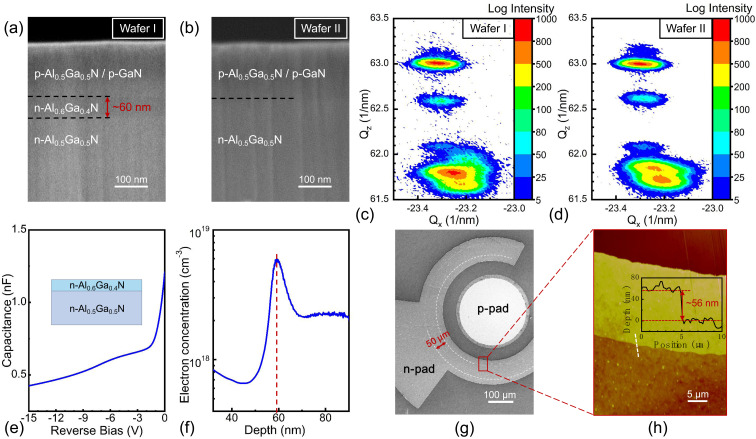
(a) and (b) are the cross-sectional SEM images of Wafer I and II. (c) and (d) are the XRD RSMs of (−105) plane of Wafer I and II. (e) and (f) are the *C*–*V* measurement result and the depth-dependent electron concentration profile of the Al_0.5_Ga_0.5_N/Al_0.6_Ga_0.4_N wafer. (g) Top-view SEM image of the fabricated Device A. (h) AFM image of the selected region in (g). The inset is the depth profile of recess edge.

Fig. 2a and b show the cross-sectional SEM images of Wafers I and II, respectively. As can be seen, there was an extra ∼60 nm insertion layer in Wafer I compared to Wafer II. To confirm the component of the insertion layer, HRXRD RSM measurements of the (−105) planes for Wafers I and II were performed, as shown in Fig. 2c and d, respectively. For Wafer I, a clear extra peak related to the AlGaN reciprocal lattice point (RLP) intensity distribution could be found, with *Q*_*z*_ and *Q*_*x*_ values of −61.9 nm^−1^ and −23.2 nm^−1^, which were not observed in Wafer II. Based on the asymmetric XRD RSM scan, the lattice parameters could be calculated by eqn (1):^31^1
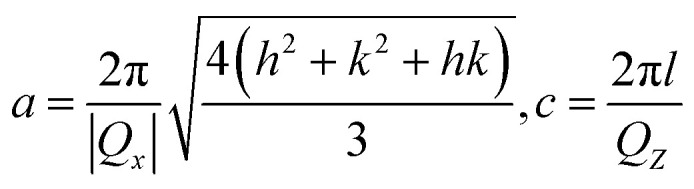


The calculated *c* and *a* values were 0.5075 and 0.3127 nm, confirming the insertion layer Al content was about 60% by Vegard's law. Furthermore, to confirm the interface electron concentration of the Al_0.6_Ga_0.4_N/Al_0.5_Ga_0.5_N heterostructure in Wafer I, a wafer the same as Wafer I was obtained except that there was no upper p-AlGaN and p-GaN, and *C*–*V* measurements were performed on it at RT. The result is shown in Fig. 2e. Accordingly, the electron concentration *N*(*x*) related to depletion width (*x*) could be obtained by eqn (2):^32^2
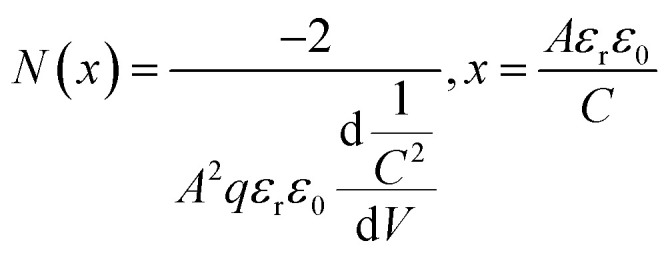
where *A* is the effective contact area (*A* = π*R*^2^, *R* = 385 μm), *q* is the elementary charge (1.602 × 10^−19^ C), *ε*_r_ is the relative dielectric constant (9.39 for AlGaN), and *ε*_0_ is the vacuum dielectric constant (8.859 × 10^−14^ F cm^−1^), respectively. Fig. 2f shows the calculated relationship between the electron concentration and the depletion width. A peak electron concentration of 6 × 10^18^ cm^−3^ occurred at ∼60 nm below the surface, exactly at the n-Al_0.6_Ga_0.4_N/n-Al_0.5_Ga_0.5_N heterostructure interface. The sheet carrier concentration of the Al_0.6_Ga_0.4_N/Al_0.5_Ga_0.5_N heterostructure could be calculated as 2.65 × 10^12^ cm^−2^. The polarization-relative parameters in the simulation below were set according to this experimental result. As for the device, the top-view SEM image for the fabricated Device A is shown in Fig. 2g, showing the recess structure with a width of ∼50 μm. To confirm the depth of the recess, AFM measurements were carried for Device A at the edge of the recess structure as marked by the red rectangle in Fig. 2g. The result is shown in Fig. 2h, from which the recess depth was estimated to be ∼56 nm.

**Fig. 3:**
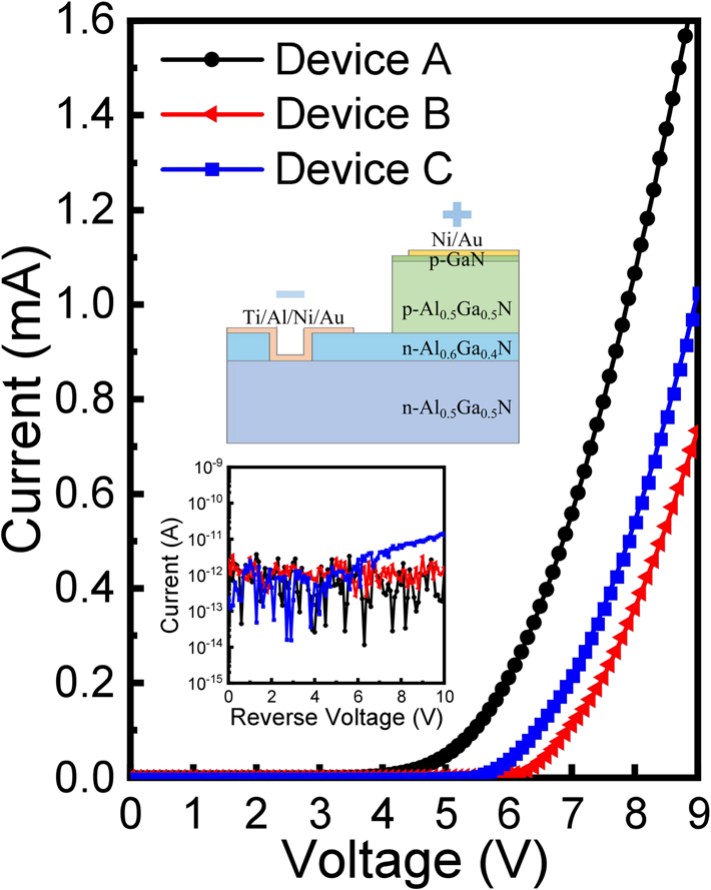
*I*–*V* curves of the Devices A, B and C. The inset indicates the electrical injection direction of the applied bias in the measurements and the *I*–*V* curves at reverse voltage.

To verify the influences of the n-Al_0.6_Ga_0.4_N/n-Al_0.5_Ga_0.5_N heterostructure and the recess structure on the electrical properties, the *I*–*V* characteristics for Devices A, B, and C were measured, as shown in Fig. 3. Obviously, the threshold voltage of Device A with the recessed n-Al_0.6_Ga_0.4_N/n-Al_0.5_Ga_0.5_N heterostructure n-contact was ∼5.6 V, which was 1.0 V lower than that of Device C (∼6.6 V), which indicated that the heterostructure and recess structure had a positive effect on the contact. Besides, it is worth noting that Device B possessed a larger threshold voltage of 0.3 V than Device C, since the n-Al_0.6_Ga_0.4_N insertion layer between the p-Al_0.5_Ga_0.5_N and n-Al_0.5_Ga_0.5_N layers induced an additional energy barrier height and increased the series resistance of the p–n junction. However, the compromised effect for the n-Al_0.6_Ga_0.4_N insertion layer and the recessed n-contact structure enabled a notable improvement of the threshold voltage for Device A, demonstrating that the recess structure played an importance role in decreasing the n-contact resistance for the Al-rich AlGaN-based p–n diodes. In addition, the inset of Fig. 3 shows the reverse leakage *I*–*V* in log scale. Thanks to the insert layer, the reverse leakages of Devices A and B stayed at a low level in 10 V reverse voltage. This was because the Al-rich n-Al_0.6_Ga_0.4_N layer led to a conduction band barrier at the n-Al_0.6_Ga_0.4_N/p-Al_0.5_Ga_0.5_N interface and a valence band barrier at the n-Al_0.5_Ga_0.5_N/n-Al_0.6_Ga_0.4_N interface. Even when the p–n junction was reversely biased at 10 V, it could still effectively block the transport of electrons and holes. On the contrary, the reverse leakage of the conventional Device C rose at about −6 V bias.

**Fig. 4:**
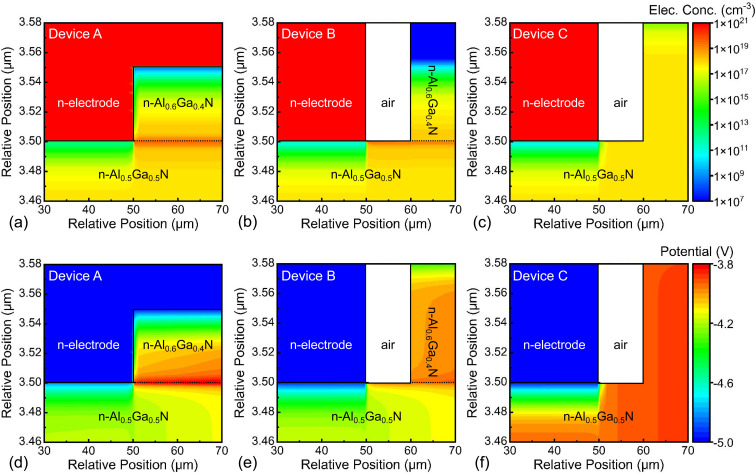
(a)–(c) Local electron concentration distribution at equilibrium and (d)–(f) potential distributions at 5 V near n-contact in Devices A, B and C.

To illuminate the effects of the heterostructure recess n-contact on the electron injection, numerical calculations for these p–n diodes were carried *via* APSYS software. Fig. 4a–c show the local electron concentration distribution around the n-electrode edge or recess edge for the fabricated p–n diodes Devices A, B, and C at equilibrium. The electron concentrations at the n-Al_0.6_Ga_0.4_N/n-Al_0.5_Ga_0.5_N heterostructure interfaces in both Devices A and B were significantly higher than that of Device C, due to the existence of polarization charges, which could increase the surface electron concentration for the n-Al_0.5_Ga_0.5_N. In particular, the electron concentration in Device A around the recess edge was also higher than that of Device B around the n-electron edge, indicating the positive effect of the recess structure. In addition, at the region 30 nm below the center of the n-electrode, the electron concentrations were 6.44 × 10^17^, 5.66 × 10^17^, and 5.35 × 10^17^ cm^−3^ for Devices A, B, and C, respectively, implying the necessity of the recess structure. To analyze the electron-transport process, potential distributions for the diodes at 5 V were calculated, as shown in Fig. 4d–f. Apparently, the MS n-contact depletion region potential differences for Devices A, B, and C successively increased, indicating the better n-contact of Device A. Besides, there existed a high potential electron-transport channel that was directly attached to the n-electrode, also benefiting the electron injection. These might be the reasons why Device A had a lower threshold voltage than Devices B and C.

**Fig. 5:**
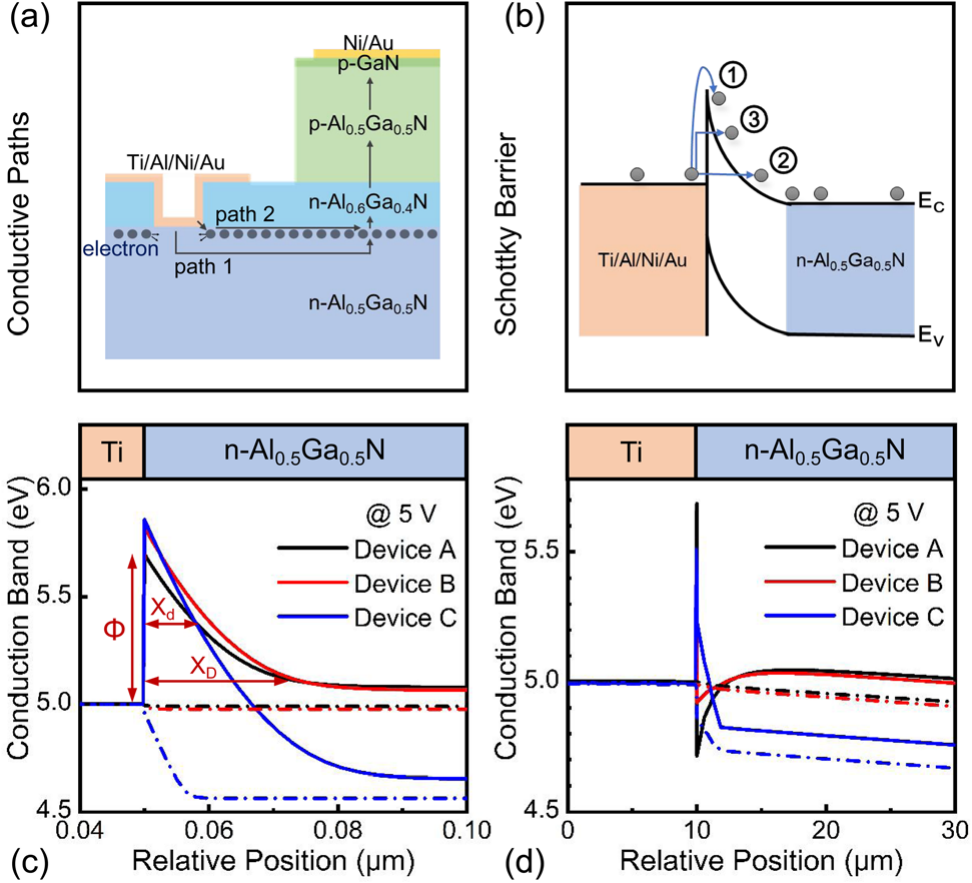
(a) Electron transport path diagram in Device A. (b) Electron transport processes at the MS contact. *E*_C_ and *E*_V_ represent the conduction and the valence bands, respectively. (c) And (d) are the calculated conduction band diagrams of metal/n-AlGaN contact along paths 1 and 2 in Devices A, B and C at 5 V, respectively.

According to the potential distribution, there may be two paths, denoted as path 1 and path 2, through which the electrons can inject into the diodes, as shown in Fig. 5a. In path 1, electrons from the cathode vertically pass through the depletion region of the MS contact and laterally transport to the p–n junction. In path 2, electrons from the cathode directly tunnel to the semiconductor through the corner of the metal close to the mesa structure and laterally transport near the semiconductor surface to the p–n junction. Owing to the direct contact of the metal electrode and the polarization-induced conductive channel, path 2 in Device A is the recess corner close to the mesa structure and the n-Al_0.6_Ga_0.4_N/n-Al_0.5_Ga_0.5_N interface.

There are generally three carrier transport processes in a MS contact: thermionic emission, intraband tunneling, and thermionic-assisted intraband tunneling, denoted as processes ①, ②, and ③, respectively, as shown in Fig. 5b. The energy band diagrams near the MS contact of Devices A, B, and C along paths 1 and 2 were calculated, as shown in Fig. 5c and d, respectively, at the voltage of 5 V, to analyze the three processes of each device. For path 1, a Schottky barrier height (*Φ*) of 0.86 eV was formed at the MS contact for Device C. By comparison, the Schottky barrier height was reduced by 0.04 eV in Device B, where an n-Al_0.6_Ga_0.4_N layer was inserted, and was further decreased by 0.12 eV in Device A, where an n-Al_0.6_Ga_0.4_N layer and a recess contact structure were both introduced. These results could be mainly attributed to the polarization-induced higher carrier concentration at the MS interface in Device A. Hence, process ① was sequentially enhanced from Device C to A. Then, the depletion widths (*χ*_D_) of the n-contact in Devices A, B, and C were 25, 27, and 16 nm, respectively, which were not short enough for process ②. Next, for process ③, within a tunneling distance of several nanometers, the tunneling distance (*χ*_d_) sequentially increased in Devices A, C, and B at the same barrier height. Therefore, Device A had both the highest thermionic emission and thermionic-assisted intraband tunneling efficiencies. For path 2, at the MS interface, the conduction band was even lower than the metal work function, which could favor the electron tunneling transport processes ② and ③. Particularly, the band diagram illustrated that Device A exhibited a larger difference between the conduction band and metal work function than Device B and that a Schottky barrier and a depletion region still existed in Device C. Consequently, the electron tunneling transport processes ② and ③ in path 2 in Devices C, B, and A were sequentially enhanced. Here, it is worth noting that it was difficult to distinguish which was the main current path because path 1 shared a larger contact area while path 2 possessed a higher tunneling probability. However, no matter which path, Device A had the strongest carrier transport processes, including both thermionic emission and tunneling, among the three p–n diode devices.

## Conclusions

In summary, we have proposed a more convenient and stable strategy to effectively optimize the metal/n-AlGaN contact by introducing a heterostructure with a polarization effect and by etching a recess structure through the heterostructure beneath the n-contact metal. As a result, a quasi-vertical Al_0.5_Ga_0.5_N p–n diode with a ∼1 V reduced forward voltage was achieved. Numerical calculations revealed that the increased surface electron concentration beneath the metal induced by the polarization effect and the recess structure were the main reasons for the reduced forward voltage. This could also decrease the Schottky barrier height as well as provide a better carrier transport channel, enhancing both the thermionic emission and tunneling processes. This investigation provides an alternative approach to obtain a good n-contact, especially for Al-rich AlGaN-based devices, such as diodes and LEDs.

## Conflicts of interest

There are no conflicts to declare.

## Supplementary Material
